# Comparative Analysis of the Physicochemical Properties and Bacterial Diversity of Cowpeas During Natural and Inoculated Fermentation in Different Provinces

**DOI:** 10.3390/microorganisms14061248

**Published:** 2026-06-02

**Authors:** Zichang Shao, Fengbo Ma, Xuanzhe Chang, Qian Liu, Yuxin Liu, Fan Xia, Xiuzhi Gao

**Affiliations:** Beijing Key Laboratory of Agricultural Product Detection and Control of Spoilage Organisms and Pesticide Residue, Beijing University of Agriculture, Beijing 102206, China; 18515990775@163.com (Z.S.); 15116938507@163.com (F.M.); ccxz1029@163.com (X.C.); 13078561157@163.com (Q.L.); lyx61525@163.com (Y.L.); 15210249078@139.com (F.X.)

**Keywords:** bacterial diversity, cowpea, high-throughput sequencing, inoculated fermentation, natural fermentation

## Abstract

Fresh cowpeas have a limited shelf life at room temperature. Fermentation of cowpeas not only preserves their nutritional value but also prolongs their shelf life. This study categorized cowpea fermentation processes into natural and inoculated methods, focusing on analyzing physicochemical indices, acid production, and bacterial diversity throughout the cowpea fermentation process. We compared the moisture, protein content, and vitamin C levels of cowpeas. The acidification process was monitored using pH, total acid, and nitrite contents as indicators. Illumina MiSeq sequencing was employed to analyze the bacterial communities in fermented cowpeas at different fermentation stages. The experimental results indicated that during fermentation, pH, total acid content, and nitrite content all changed significantly. *Lactobacillus* exhibited high dominance in both natural fermentation and inoculated fermentation processes. Moreover, under inoculated fermentation conditions, its population size was significantly greater than that in natural fermentation. Analysis of bacterial community composition revealed that microbial diversity tended to decrease with prolonged fermentation time in both natural and inoculated fermentation systems. The results demonstrate that inoculation fermentation can shorten the fermentation cycle, lower nitrite levels, and confirm that lactic acid bacteria are the dominant microbial genus in vegetable fermentation.

## 1. Introduction

Fermented vegetables have a long history in China, dating back to the Zhou Dynasty. Using vegetables as the raw material, these products undergo natural fermentation by yeast and lactic acid bacteria (LAB), resulting in the formation of fermented vegetable products [1]. Initially, fermentation was carried out to extend the shelf life of vegetables. However, with societal development, fermentation is no longer merely for the effective preservation of vegetables [2]. The convenient production process endows fermented vegetables with a unique flavor and texture. Moreover, it reduces production costs, utilizes abundant raw materials, and promotes digestion, which makes consumers more inclined to choose fermented vegetables [3].

Cowpea (*Vigna unguiculata*), a widely cultivated legume crop in China, is processed into fermented products through lactic acid bacteria fermentation. It is celebrated for its fresh, savory flavor and crispy texture, along with bacteriostatic, anti-cancer, and anti-aging properties [4,5]. Currently, homemade fermentation remains the primary method for producing fermented cowpea in China. However, the inability to control environmental factors leads to prolonged fermentation cycles, unstable product quality, and contamination by harmful microorganisms [6].

During homemade cowpea fermentation, the species and quantity of microorganisms in the fermentation system are key factors influencing product safety. These microorganisms are governed by multiple factors, including raw materials, temperature, fermentation duration, and solute concentration, which together drive variations in dominant bacterial communities and flavor compounds across different fermented vegetable products [7,8]. *Lactiplantibacillus plantarum* is one of the most widely used lactic acid bacteria in fermented vegetables. It exhibits strong adaptability to legume matrices, high acid-producing capacity, and excellent antimicrobial activity, making it an ideal starter culture for cowpea fermentation. A study by Oyedoh demonstrated that LAB fermentation of cowpea enhances nutrient content and improves flavor profiles [9]. Nevertheless, LAB may not consistently dominate the fermentation process; thus, it is necessary to screen beneficial microorganisms for targeted application in cowpea fermentation.

Fresh cowpeas have a relatively short shelf life at room temperature and are susceptible to damage or spoilage during transportation. However, fermented vegetables, besides offering high nutritional value, can be stored for extended periods under ambient conditions. This study primarily compared the pH, acidity, nitrate content, and bacterial diversity of cowpeas from three provinces (Guangdong, Hebei, and Hunan) subjected to natural fermentation (NF) and inoculated fermentation (IF), analyzed the relationships among these parameters, and explored potential applications in functional foods.

These findings provide a scientific foundation for the development of novel functional food products derived from cowpea fermentation.

## 2. Materials and Methods

### 2.1. Bacterial Culture and Activation

*Lactobacillus plantarum* NL5 (*Lactiplantibacillus plantarum* strain 2979, MT611912) and *Lactobacillus* LV53 (*Lactiplantibacillus* sp., HQ259239.1) were isolated from traditional fermented vegetables in Shanxi Province. Currently, the strains are deposited at the Microbial Comprehensive Control Laboratory of the Food Science and Engineering College, Beijing Agricultural University. The bacterial strains are stored in 50% (*v*/*v*) glycerol at −80 °C under cold storage conditions. When culturing these strains separately in MRS broth (Land Bridge, Beijing, China), they were incubated at 37 °C for 12 h to reach a bacterial count of approximately 8–9 log CFU/mL [10].

### 2.2. Preparation of Fermented Cowpeas

Fresh cowpeas were procured from Huailai County, Zhangjiakou City, Hebei Province; Panyu District, Guangzhou City, Guangdong Province; and Huarong County, Yueyang City, Hunan Province. The fresh, mildew-free, and undamaged cowpeas were cleaned and then blanched for about 10 s. The blanched cowpeas were transferred to 4 °C cold water for cooling. Once cooled, the cowpeas are cut into 1–2 cm segments. These segments are subsequently immersed in 1.5% salt water (*w*/*v*) for 1 h to draw out moisture. After that, the cowpeas are packed and then submerged in Sichuan pepper water (the volume ratio of Sichuan pepper water to cowpeas is 6:5). The ingredients for the Sichuan pepper water include 1.5% salt, 0.3% Sichuan pepper, 0.2% star anise, and 0.3% ginger slices.

After being cooled again, the cowpeas are treated differently:

(1) NF: Injected with 0.5% sterile deionized water.

(2) IF: Injected with 0.5% of a pre-prepared fermentation solution, which had a total bacterial count of 8–9 log CFU/mL and a ratio of NL5 to V53 of 2:1.

The mixture was then uniformly mixed and sealed in an incubator maintained at 25 °C for 4 days. Each time, 10.0 g of the sample (liquid/solid = 1:1) was taken for analysis using a breakage machine (SPJ002S, SUPOR, Hangzhou, China). The sample was rapidly agitated at room temperature for about 10 s until it formed a paste.

### 2.3. Changes in the Physicochemical Indexes of Cowpeas from Different Provinces

Moisture was determined by weighing 30.0 g of the sample, freeze-drying it for 24 h, and calculating the weight difference. Vitamin C content was analyzed using a vitamin C content test kit (Nanjing JianCheng Bioengineering Institute, Nanjing, China). Protein content was measured with a BCA protein concentration assay kit (Solarbio, Beijing, China). Both vitamin C and protein content were quantified using a UV–Vis spectrophotometer (UV759CRT, Yoke Instrument, Shanghai, China).

The pH level of the samples was determined with a pH meter (FE28, FiveEasy Plus, Shanghai, China). The total acid determination was slightly modified with reference to Fu [11]. A total of 25 g of the sample was weighed, with a solid/liquid ratio of 1:1, and ground in a mortar. Then, the sample was transferred to a 250 mL volumetric bottle, and the volume was fixed. The liquid to be measured was quickly filtered using filter paper and then titrated. Total acid was determined using acid–base indicator titration. The content of nitrite was determined by the ethylenediamine hydrochloride method and slightly modified according to Chen [12].

### 2.4. Preparation of Bacterial Diversity During Fermentation of Cowpeas from Different Provinces

Samples of cowpeas originating from Guangdong, Hebei, and Hunan provinces were subjected to both natural fermentation and inoculum fermentation processes. After labeling the cowpea fermentation samples, the dynamic changes in microbial indices during a four-day fermentation period were recorded. Samples taken on days 0, 1, 2, 3, and 4 were subjected to microbial diversity analysis.

### 2.5. Microbial Genome Extraction

According to the specifications of the E.Z.N.A.^®^ soil kit (Omega Bio-tek, Norcross, GA, USA), the total DNA (Axygen Biosciences, Union City, CA, USA) was extracted, the concentration and purity of DNA were detected, and the quality of DNA was examined using 1% agarose gel electrophoresis. The variable region of V3–V4 was amplified using PCR with 338F (5′-ACTCCTACGGAGGAGCAGCAGCAGCAGCAG-3′) primers and 806 R (5′-GGACTACHVGGGTCTAAT-3′) primers. Primers were purchased from Sangon Biotech Co., Ltd., Beijing, China.

The amplification procedure was as follows: predenaturation at 95 °C for 3 min, 27 cycles (denaturation at 95 °C for 30 s, annealing at 55 °C for 30 s, extension at 72 °C for 30 s), and a final extension of 10 min at 72 °C.

The amplification system was 20 μL, 4 μL 5 × FastPfu buffer, 2 μL 2.5 mM dNTPs, 0.8 μL primer (5 μM), 0.4 μL FastPfu polymerase, 10 ng DNA template [12].

### 2.6. High-Throughput Sequencing

PCR products were recovered using a 2% agarose gel, purified, eluted using Tris-HCl, and detected using 2% agarose gel electrophoresis. Sequencing was carried out on the Illumina MiSeq Miseq PE300 platform (Illumina, San Diego, CA, USA) by Shanghai Major Biopharmaceutical Technology Co., Ltd. (Shanghai, China). The original data have been uploaded to the NCBI database.

### 2.7. OTU Clustering and Taxonomic Analysis

After assembling the raw sequences using FLASH (v1.2.11) software, OTU clustering was performed on sequences with a similarity threshold of 97%, using UPARSE (V 7.0.1090) software [13,14]. The sequences annotated as chloroplast and mitochondrial were removed from all samples. The number of sequences per sample was normalized to 20,000. Despite normalization, the average sequence coverage (Good’s coverage) for each sample remained at 99.09%. Using the RDP (v2.11) classifier, OTU-based species annotations were generated by comparing the sequences against the Silva 16S rRNA gene database with a confidence threshold of 70% [15]. The community composition at different taxonomic levels was then analyzed for each sample.

### 2.8. Data Analysis

SPSS 27.0, Origin 2024, and MajorbioCloud 2024 software were used for data analysis and chart drawing. All data were subjected to three replicate tests.

## 3. Results

### 3.1. Physicochemical Index Results

**Figure 1:**
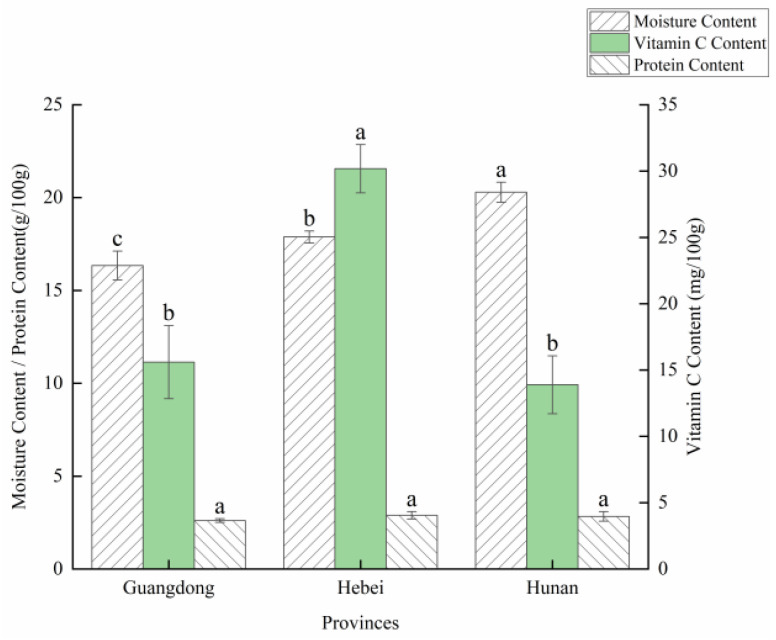
Content of moisture, vitamin C, and protein in raw cowpeas from different provinces (day 0). Different lowercase letters (a, b, c) indicate significant differences at the *p* < 0.05 level.

The moisture, VC (vitamin C), and protein contents of cowpea samples from different provinces are presented in Figure 1. The cowpea sample from Hunan Province exhibited the highest moisture content at 20.29 ± 0.54 g/100 g, while the Guangdong Province sample had the lowest at 16.34 ± 0.78 g/100 g. The moisture contents varied significantly between the three provinces (*p* < 0.05). For VC content, the sample from Hebei Province showed the highest level at 30.18 ± 1.82 mg/100 g, which was significantly higher than those from Guangdong (15.6 ± 2.76 mg/100 g) and Hunan (13.89 ± 2.18 mg/100 g) provinces (*p* < 0.05). In terms of protein content, although variations were observed among provinces, no significant differences were detected (*p* < 0.05). The lowest protein content was found in the sample from Guangdong Province (2.61 ± 0.10 g/100 g), while the highest was observed in the sample from Hebei Province (2.89 ± 0.21 g/100 g).

**Figure 2:**
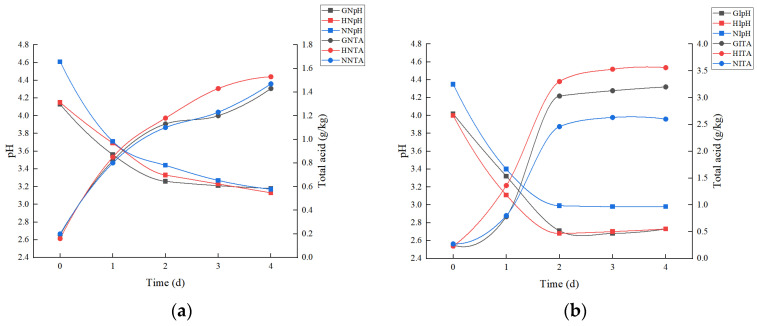
Changes in pH and total acid during 4 days of fermentation of cowpeas from different provinces. (**a**): Natural fermented cowpeas. (**b**): Inoculated fermented cowpeas. Group names: first letter = province (G: Guangdong, H: Hebei, N: Hunan), second letter = fermentation type (N: natural, I: inoculated), TA: total acid.

Lactic acid bacteria (LAB) produce organic acids during their growth and metabolism. Figure 2 indicates that across fermented cowpea samples from the three provinces, pH exhibited a decreasing trend while total acid content increased, an inverse correlation with pH changes. Compared with natural fermentation (NF), inoculated fermentation (IF) displayed more stable trends in total acid content and pH dynamics. Specifically, after 4 days of fermentation, for NF, pH ranged from 3.0 to 3.5 across the three provinces, and total acid content ranged from 1.40 to 1.60 g/kg. For IF, pH levels across all provinces were consistently lower (range of 2.7–3.0), and total acid content was higher. Notably, significant differences in total acid content were observed among provinces under IF; the lowest level was recorded in Hunan (2.63 g/kg) and the highest in Hebei (3.81 g/kg). This acid accumulation is driven by LAB, which convert carbohydrates in cowpeas into lactic acid and other organic acids under anaerobic conditions, thus reducing pH and increasing total acid content in fermented cowpeas [16].

Nitrite content is a critical safety indicator for fermented vegetables. China’s National Food Safety Standard specifies a maximum allowable limit of 20 mg/kg for nitrites (expressed as NaNO_2_) in pickled vegetables [17]. To assess the safety of fermented cowpeas under different processing conditions, natural fermentation (NF) and inoculated fermentation (IF) were applied to cowpea samples collected from Guangdong, Hebei, and Hunan provinces.

**Figure 3:**
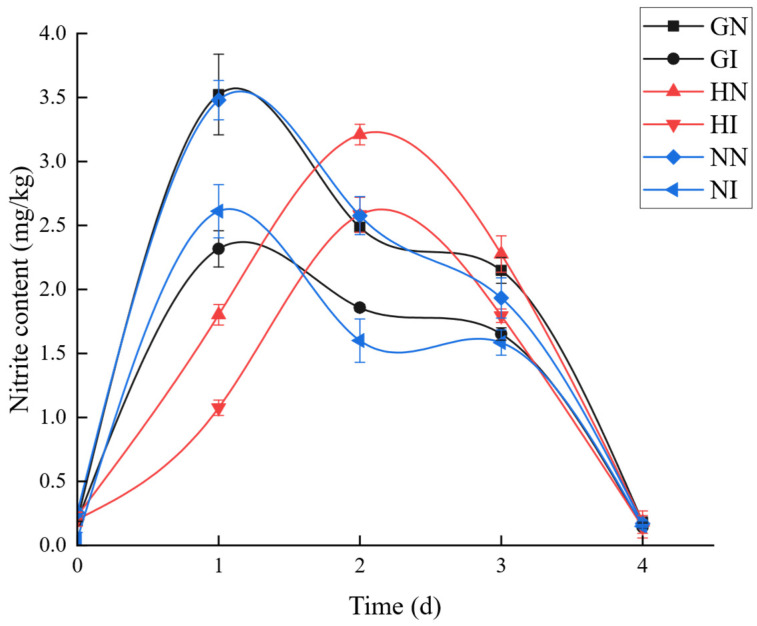
Changes in nitrite content during 4 days of fermentation of cowpeas from different provinces. Group names: first letter = province (G: Guangdong, H: Hebei, N: Hunan), second letter = fermentation type (N: natural, I: inoculated).

As shown in Figure 3, nitrite content dynamics during fermentation varied across the three provinces. Nitrite levels in both naturally fermented (NF) and inoculated fermentations (IF) cowpea samples remained below the 20 mg/kg safety limit throughout fermentation, and decreased to <0.2 mg/kg by day 4. For both NF and IF, nitrite levels peaked on day 2 in Guangdong and Hunan cowpeas, whereas in Hebei, peaks occurred on day 3. Among NF groups, the highest nitrite peak was observed in Hunan cowpeas (3.52 mg/kg). Subsequently, nitrite levels in this group gradually declined to 0.18 mg/kg by day 4. Under identical fermentation conditions, IF groups exhibited lower peak nitrite levels than their NF counterparts. The largest difference in peak nitrite levels between NF and IF was observed in Guangdong cowpeas (1.21 mg/kg). This difference was statistically significant relative to the corresponding differences in Hebei and Hunan cowpeas (*p* < 0.05).

### 3.2. Bacterial Colony Diversity Analysis

**Figure 4:**
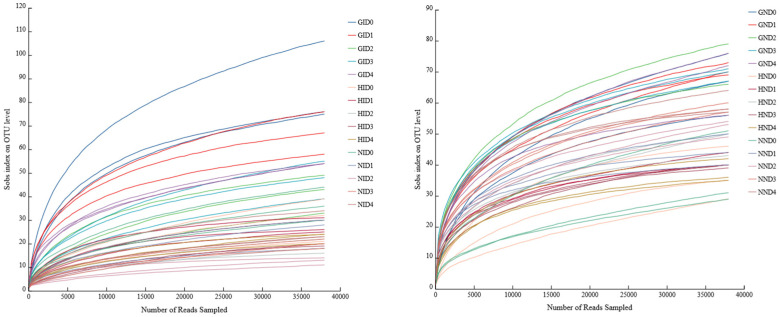
Dilution curve for bacterial colonies in fermented cowpeas. Group names: first letter = province (G: Guangdong, H: Hebei, N: Hunan), second letter = fermentation type (N: natural, I: inoculated), D0: 0 d, D1: 1 d, and so on.

Dilution curves are a standard tool for comparing species richness across samples with varying sequencing depths. A plateaued curve indicates that the sequencing depth is sufficient to capture the majority of species present in the sample. Figure 4 shows the bacterial community dilution curves for fermented cowpea samples from Guangdong, Hebei, and Hunan provinces during fermentation. As sequencing depth increases, each curve tends to stabilize, suggesting that the sequencing data for the microbial communities of fermented cowpeas are sufficient to accurately reflect their species richness.

From the DNA sequencing of 90 fermentation cowpea samples, a total of 5,123,334 effective bacterial sequences were obtained, with an average length of 421 bp. OTU-based Alpha diversity analysis revealed that sequencing coverage across all samples ranged from 99.91% to 99.98%, confirming that the sequencing data sufficiently captured the microbial diversity in the samples. Table 1 and Table 2 summarize key metrics of bacterial community richness and diversity: the Ace index, Chao index, and Sob index (which quantify community richness), as well as the Shannon index and Simpson index (which assess community diversity).

The bacterial community composition during the natural fermentation process of cowpeas is summarized in Table 1. In cowpeas from Guangdong province, the bacterial community richness index reached a peak value of 88.40 on day 2, which is higher than the corresponding values in samples from Hebei and Hunan provinces. For Guangdong cowpeas, the diversity trend (as measured by Shannon/Simpson indices) initially increased and then declined. In Hebei cowpeas, both bacterial community richness and diversity exhibited a similar rise-and-fall pattern over the fermentation time. By contrast, Hunan cowpeas’ bacterial community richness showed a sustained increasing trend throughout the process. These findings suggest that bacterial communities undergo significant succession during the fermentation process of cowpeas. Across all three provinces, the Shannon and Simpson indices of bacterial communities displayed an inverse relationship, which aligns with their distinct ecological roles—Shannon quantifies combined species richness and evenness, while Simpson reflects community dominance (higher Simpson values indicate greater dominance by a few species).

Table 2 summarizes changes in bacterial diversity and community richness indices during the initial stage of cowpea fermentation. Across Guangdong, Hebei, and Hunan provinces, bacterial communities exhibited peak diversity on day 1. This is likely due to the highly abundant nutrients in the initial fermentation system, which support rapid bacterial proliferation and thereby enhance community richness indices. Following day 1, bacterial diversity in all three provinces first decreased and then recovered. These temporal dynamics confirm that significant bacterial succession takes place during cowpea fermentation.

### 3.3. Characteristics of Bacterial Community During Fermentation of Cowpeas

**Figure 5:**
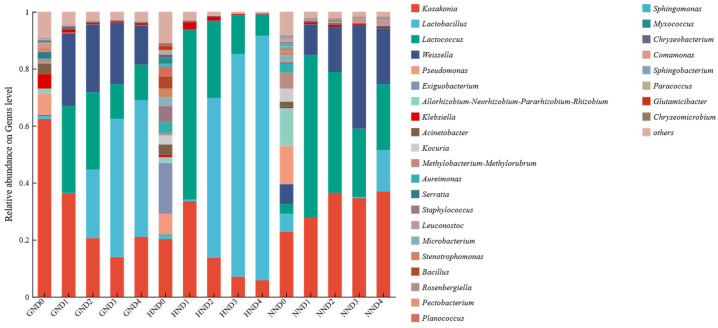
The abundance of bacterial genera in naturally fermented cowpea samples at the genus level. GN: Guangdong natural fermentation; HN: Hebei natural fermentation; NN: Hunan natural fermentation; D: day, the number after D represents the fermentation days.

The structure of bacterial communities during the fermentation process of cowpea is shown in Figure 5. The most diverse bacterial community was observed in naturally fermented cowpea samples on day 0, with the genus *Kosakonia* accounting for the highest relative abundance. As fermentation progressed, in samples from Guangdong province, dominant bacterial genera included *Kosakonia*, alongside *Weissella*, *Lactococcus*, and *Lactobacillus*; in the Hebei province samples, the dominant community shifted to *Kosakonia*, *Lactococcus*, and *Lactobacillus*; in the Hunan province samples, the bacterial community was similar to Guangdong’s, though *Lactobacillus* was only detected in Hunan’s final fermented product. Across all three provinces, the core bacterial communities primarily consisted of *Lactobacillus*, *Lactococcus*, and *Weissella*, consistent with the findings of [18].

After two days of fermentation, the proportion of *Lactobacillus* increased significantly in cowpeas from both Guangdong and Hebei provinces, reaching 24.07% and 56.06%, respectively. Subsequently, in Guangdong cowpeas, the proportion of *Lactobacillus* was approximately 48.27%. In Hebei cowpeas, it continued to rise, reaching 85.77% by day 4 and establishing itself as the absolute dominant bacterial genus. In Hunan cowpeas, *Lactobacillus* was only detected in the final product, accounting for a low proportion of 14.49%, whereas the proportion of *Kosakonia* remained relatively stable throughout the fermentation process, ranging from 22.86% to 36.96%.

Kosakonia possesses multifunctional metabolic capabilities that allow it to grow under a broad range of temperatures, pH levels, and salt concentrations [19]. Additionally, it inhibits fungal mycelial growth during fermentation by producing volatile metabolites, including terpenoids, ketones, alcohols, fatty acid derivatives, and ethers [20].

**Figure 6:**
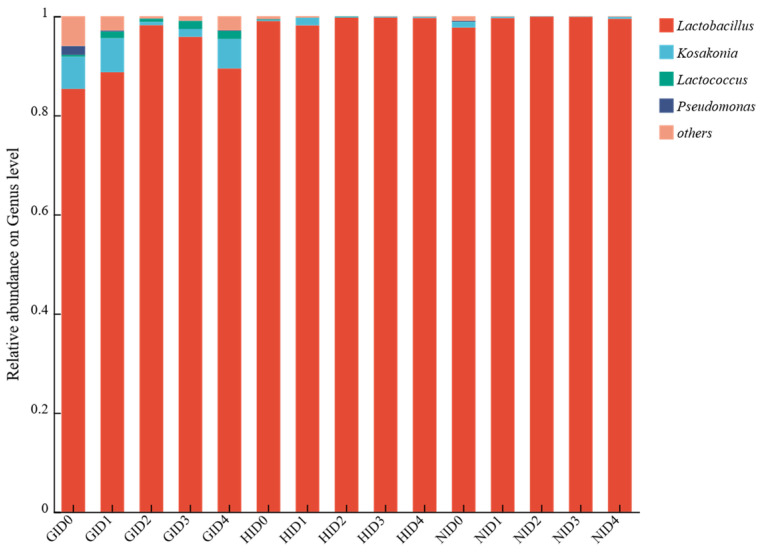
The abundance of bacterial genera in inoculated fermented cowpea samples at the genus level. GI: Guangdong inoculated fermentation; HI: Hebei inoculated fermentation; NI: Hunan inoculated fermentation; D: day, the number after D represents the fermentation days.

The structure of the bacterial community associated with cowpea fermentation, inoculated with *Lactobacillus*, is shown in Figure 6. Compared to natural fermentation, the intentional inoculation of *Lactobacillus* results in the *Lactobacillus* genus becoming the dominant bacterial genus from day 0 onwards. As fermentation progresses, the abundance of *Lactobacillus* in cowpeas from Guangdong province first increases and then decreases, peaking on day 2 before gradually declining, although it remains the dominant bacterial genus in the fermentation system. In contrast, there are no significant differences in bacterial community succession or the dynamics of dominant bacterial groups during fermentation between cowpeas from Hebei and Hunan provinces, and *Lactobacillus* remains the absolute dominant bacterial genus throughout the process.

Liu demonstrated that post-inoculation changes in microbial community improve the acidity and flavor quality of Hunan bean soup [21]. Their study indicated that artificial inoculation of LAB effectively controls fluctuations in dominant strains during cowpea fermentation across different provinces.

**Figure 7:**
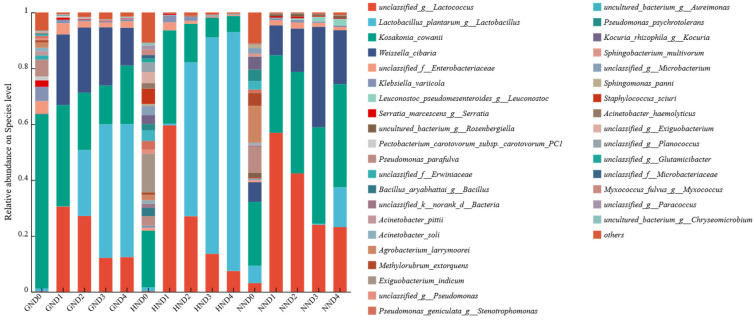
The abundance of bacterial genera in naturally fermented cowpea samples at the species level. GN: Guangdong natural fermentation; HN: Hebei natural fermentation; NN: Hunan natural fermentation; D: day, the number after D represents the fermentation days.

At the species level, the trends in naturally fermented cowpea samples are illustrated in Figure 7 and are analogous at the genus level. The fermentation system is dominated by four key bacterial species: unclassified Lactococcus, *Lactobacillus plantarum*, *Kosakonia cowanii*, and *Weissella cibaria*. At the start of fermentation (day 0), neither *Lactococcus* nor *Lactobacillus plantarum* held dominant status; however, under favorable fermentation conditions, both species grew rapidly and became the dominant taxa on day 1 in the samples from Guangdong and Hebei provinces. In Hunan cowpeas, *Lactobacillus plantarum* made up 6.22% of the bacterial population on day 0. Subsequently, Lactococcus bacteria proliferated rapidly, peaking at 56.91% on day 1 before gradually decreasing to 23.16% by day 4, after which its abundance stabilized—at this point, *Lactobacillus plantarum* had decreased to 14.29%.

**Figure 8:**
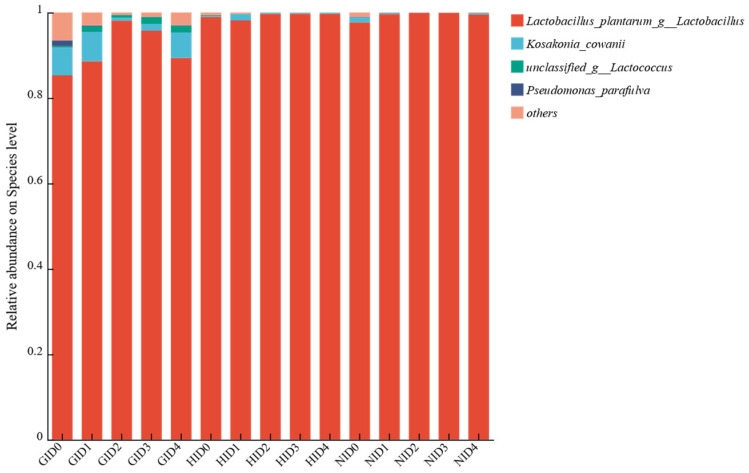
The abundance of bacterial genera in inoculated fermented cowpea samples at the species level. GI: Guangdong inoculated fermentation; HI: Hebei inoculated fermentation; NI: Hunan inoculated fermentation; D: day, the number after D represents the fermentation days.

The bacterial community changes in inoculated fermented cowpea samples are shown in Figure 8, which mirror the trends at the genus level. At the early stage of fermentation, *Lactobacillus plantarum* was the predominant species in inoculated fermented cowpea samples. Over the first 24 h of fermentation, its abundance gradually increased, making it the absolute dominant bacterium on day 1.

**Figure 9:**
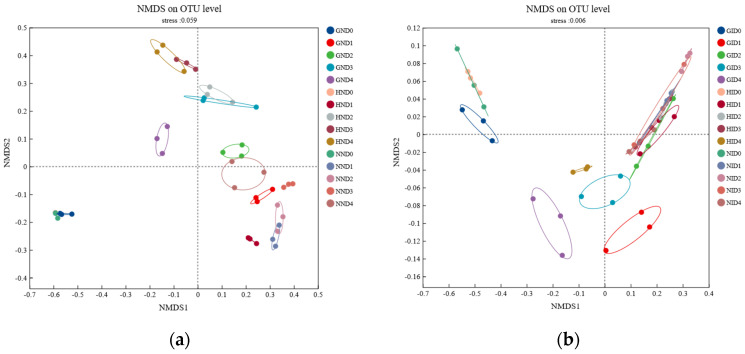
NMDS analysis of bacterial communities in fermented cowpeas at the OTU level. (**a**): Naturally fermented cowpeas. (**b**): Inoculated fermented cowpeas. Group names: first letter = province (G: Guangdong, H: Hebei, N: Hunan), second letter = fermentation type (N: natural, I: inoculated), D: day, the number after D represents the fermentation days.

Based on OTU-level NMDS analysis, as shown in Figure 9,the bacterial communities of fermented cowpea samples from Guangdong and Hunan provinces exhibited distinct separation along the second axis relative to those collected on days 1, 2, and 3. For Hebei province samples, the bacterial communities at days 0 and 1 of fermentation showed significant differences relative to those at days 2, 3, and 4. In Guangdong province samples, the bacterial community structure changed significantly throughout fermentation, with only the day 2 sample displaying minor differences when compared to samples from other provinces. For Hunan province samples, the bacterial communities at days 0 and 4 showed distinct separation along the second axis relative to those at days 1, 2, and 3, while the communities at days 1, 2, and 3 exhibited overlapping clustering patterns with minimal differences. These clustering trends were consistent with the results from the Alpha diversity index and microbial community analyses, indicating that prolonged fermentation time induces changes in the microbial community structure of fermented cowpeas.

### 3.4. Comparison of Bacterial Community Compositions During the Cowpea Fermentation Process

Venn diagram-based data analysis revealed the shared and unique bacterial operational units (OTUs) across the fermentation processes of cowpea samples from different provinces.

**Figure 10:**
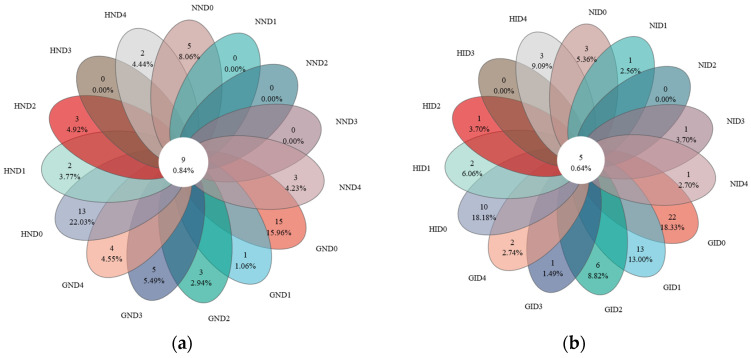
Venn diagram of fermented cowpea bacteria in different provinces. (**a**): Natural fermented cowpeas. (**b**): Inoculated fermented cowpeas. Group names: first letter = province (G: Guangdong, H: Hebei, N: Hunan), second letter = fermentation type (N: natural, I: inoculated),D: day, the number after D represents the fermentation days.

As shown in Figure 10a, among 15 natural fermentation samples from various provinces, a total of fifty-six bacterial OTUs were identified, with nine OTUs shared across all provinces. At the onset of fermentation, the number of province-specific OTUs was relatively high: fifteen in Guangdong, thirteen in Hebei, and five in Hunan. As fermentation progressed, the number of unique OTUs decreased gradually, leading to greater overlap between fermentation systems until fermentation concluded.

Similarly, as illustrated in Figure 10b, sixty-six bacterial OTUs were identified across 15 inoculated fermented cowpea samples from various provinces, with five OTUs shared across all provinces. Across the fermentation process, the number of province-specific OTUs was notably higher: twenty-two in Guangdong, ten in Hebei, and three in Hunan.

During cowpea fermentation, the dominant microbial species exhibit relatively minimal variability regardless of the fermentation methods used. The predominant bacterial genera detected in natural fermentation include *Lactobacillus*, *Weissella*, *Lactococcus*, and *Kosakonia*, among others.

LAB play an indispensable role in food fermentation, especially in vegetable fermentation [22]. These bacteria inhibit pathogenic microbes and exhibit biological preservative potential [23]. Furthermore, certain LAB exhibit proteolytic and lipolytic activities, facilitating the production of peptide fragments, free amino acids, and free fatty acids—key flavor compounds in fermented vegetables [24]. Upon artificial inoculation, *Lactobacillus* species rapidly emerge as the dominant microbial genus during cowpea fermentation.

## 4. Discussion

The initial microbial community on vegetable surfaces is one of the key determinants of the quality and flavor of fermented vegetables. Moreover, fermentation methods, seasonal variations, and the geographical origin of raw materials also exert an influence on the physicochemical properties and microbial composition of fermented vegetables. To investigate the impacts of different fermentation methods and regional variations on the fermentation process of cowpeas, a comparative experiment was conducted using cowpeas from Guangdong, Hebei, and Hunan provinces to assess NF and IF fermentation techniques.

Research findings indicate that cowpeas subjected to different fermentation methods exhibit distinct physicochemical variations during fermentation. Among these, pH, total acidity, and nitrite content demonstrate the most pronounced changes throughout the fermentation process. In the IF group, exogenously added LAB rapidly established dominance, thus dictating the fermentation process. Consequently, the rate of pH decline was significantly faster than in the NF group, eventually leading to a markedly higher total acidity. This phenomenon is highly consistent with research findings regarding microbial community structure and succession patterns [25]. In the NF system, the progressive increase in acidity and salinity created strong selective pressure, enabling acid- and salt-tolerant bacteria to gradually become dominant and suppress the growth of microorganisms with low acid and salt tolerance [26]. LAB metabolized soluble carbohydrates in the cowpeas to produce organic acids such as lactic acid, thereby gradually reducing the system pH and increasing the total acid content [27]. Under identical fermentation conditions, raw cowpea materials from Guangzhou, Hunan, and Hebei exhibited significant differences in physicochemical indicators after fermentation. This phenomenon is strongly linked to regional variations in climate and soil conditions among these three locations. Hebei has a larger diurnal temperature range, thus promoting carbohydrate accumulation in the cowpeas. Concurrently, raw materials from this area showed higher vitamin C contents. The combined effect of these factors enhances the metabolic activity of lactic acid bacteria, leading to accelerated lactic acid production. Consequently, pH decreases more rapidly, resulting in a significant increase in total acid content.

Both natural fermentation and inoculum fermentation exhibited a high dominance of the *Lactobacillus* genus, with a significantly higher proportion observed in inoculum fermentation compared to natural fermentation. Additionally, analysis of bacterial community composition revealed that microbial diversity gradually decreases as fermentation time extends, with a more pronounced reduction in diversity noted under inoculum fermentation conditions relative to natural fermentation.

Using Illumina MiSeq sequencing technology, Venn diagrams and heatmaps were employed for cluster analysis to discern differences in bacterial diversity and community structure between NF and IF cowpeas. Both fermentation methods showed LAB as the dominant and core microbial genus in the late fermentation phase, which aligns with the findings of Yuan [28]. Previous research has demonstrated that *Lactobacillus*, *Lactococcus*, and *Weissella* are the predominant genera in vegetable fermentations [29,30]. Apart from microbial composition, fermentation techniques also lead to divergence in bacterial metabolic functions. Relevant research indicated that different fermentation processes can enrich distinct metabolic pathways, including amino acid metabolism and carbohydrate metabolism [31]. Thierry conducted a meta-proteomic analysis of microbial communities in 75 homemade fermented vegetables using 16S rRNA gene-targeted metagenomic approaches and identified LAB as the dominant bacteria in most samples [32]. The fermentation results of cowpeas from the three provinces revealed that *Lactobacillus* dominated all samples across different bacterial groups. Chen reported that during the fermentation of local vegetables in Shanxi, *Lactobacillus*, *Lactococcus*, and *Weissella* were the predominant microbial communities, consistent with the findings of this study [12].

The changes in pH and total acidity over the course of cowpea fermentation progress were consistent with expectations. In the fermentation process, bacteria with high acidity and salt tolerance gradually become dominant, while microorganisms with low acid and salt resistance are increasingly inhibited [26]. LAB metabolized carbohydrates in cowpea samples to produce lactic acid, thereby gradually decreasing the product’s pH and increasing total acidity [27]. In IF, LAB rapidly became the dominant microbial community, thus taking a leading role in the fermentation, a trend consistent with findings on microbial community structures and succession patterns [25].

The OTU index, Shannon index, pH values, and total acid levels of fermented vegetables are correlated. During fermentation, the nitrite peak in the IF group is lower than that in the NF group, indicating that the IF is more safely controllable. It is well known that LAB can reduce nitrosamine levels [33]. Under low-pH acidic conditions (pH < 4), both the growth of nitrosamine-producing Gram-negative bacteria and the chemical decomposition of nitrosamines are inhibited, resulting in a rapid decrease in nitrosamine levels and improved food safety [34]. Following IF inoculation, *Lactobacillus* dominates the fermentation process, and the enzymes produced by its metabolism reduce the accumulation of nitrosamines, thereby lowering the nitrite peak [35]. Kim found that LAB exert a direct effect on reducing N-nitrosymethylamine (NDMA) and its precursors [36].

In summary, the bacterial diversity in natural fermentation is higher than that in inoculated fermentation. Interestingly, through analyzing the differences in bacterial communities between the NF and IF groups, as well as nitrosamine level variations between the two fermentation methods, we have demonstrated that the metabolic dynamics of the microbial communities in the two groups exhibit significant differences. Based on pH value, total acid levels, and nitrosamine changes, inoculation fermentation proves to be safer and more stable in terms of product quality, where LAB act as the dominant bacterial strain throughout the process. In conclusion, we have provided new scientific insights into bacterial diversity and its impact on nitrosamine changes in fermented cowpea products, thereby laying the foundation for the standardization of the fermentation process of cowpea products in the food industry.

## 5. Conclusions

This study investigated the microbial diversity of fermented cowpeas from Guangdong, Hebei, and Hunan provinces by comparing natural fermentation and inoculation fermentation processes. Results showed that pH, total acid, and nitrite contents changed significantly throughout the fermentation process. *Lactobacillus* exhibited a dominant presence in both NF and IF. Notably, the abundance of *Lactobacillus* in IF was significantly higher than that in NF. Analysis of bacterial community composition revealed that as fermentation time prolonged, microbial diversity gradually decreased in both NF and IF, with a more pronounced reduction observed under IF conditions.

The results showed that there were significant differences in microbial diversity in cowpeas from different provinces under both NF and IF conditions, and the dominant role of *Lactobacillus* as the primary strain was further confirmed. These findings provide a critical scientific basis for a deeper understanding of microbial ecology in vegetable fermentation.

## Figures and Tables

**Table 1:** Statistical analysis of Illumina sequencing data for bacteria in naturally fermented cowpea samples.

Samples	Ace	Chaos	Coverage (%)	Shannon	Simpson	Sobs
GND0	89.38 ± 22.84	80.74 ± 16.68	99.95 ± 0.01	0.27 ± 0.08	0.92 ± 0.03	66.33 ± 8.17
GND1	91.36 ± 12.46	83.56 ± 4.95	99.95 ± 0.01	1.63 ± 0.04	0.25 ± 0.00	70.67 ± 1.70
GND2	91.98 ± 9.83	88.40 ± 8.10	99.95 ± 0.01	1.65 ± 0.05	0.19 ± 0.00	75.67 ± 6.94
GND3	83.84 ± 1.54	80.91 ± 2.36	99.95 ± 0.00	1.38 ± 0.01	0.28 ± 0.03	69.33 ± 1.70
GND4	91.74 ± 21.20	84.58 ± 16.03	99.95 ± 0.01	1.59 ± 0.02	0.27 ± 0.01	68.00 ± 8.64
HND0	72.01 ± 37.51	46.55 ± 6.56	99.97 ± 0.01	0.08 ± 0.02	0.98 ± 0.00	36.67 ± 7.04
HND1	48.45 ± 7.05	50.30 ± 9.42	99.98 ± 0.01	1.23 ± 0.01	0.40 ± 0.01	41.33 ± 1.89
HND2	65.16 ± 15.00	57.64 ± 7.51	99.97 ± 0.01	1.38 ± 0.08	0.33 ± 0.02	47.67 ± 3.30
HND3	45.79 ± 1.37	43.30 ± 1.91	99.98 ± 0.01	1.06 ± 0.05	0.49 ± 0.02	39.67 ± 0.47
HND4	42.38 ± 3.18	41.98 ± 2.49	99.98 ± 0.00	0.93 ± 0.15	0.54 ± 0.07	37.67 ± 3.10
NND0	82.45 ± 6.18	58.07 ± 8.76	99.96 ± 0.01	0.19 ± 0.08	0.94 ± 0.03	37.00 ± 9.93
NND1	50.77 ± 5.63	51.94 ± 5.41	99.98 ± 0.01	1.23 ± 0.07	0.41 ± 0.04	44.67 ± 4.11
NND2	72.88 ± 13.13	67.43 ± 9.82	99.96 ± 0.01	1.40 ± 0.08	0.33 ± 0.03	52.00 ± 2.16
NND3	69.07 ± 2.44	67.24 ± 0.87	99.97 ± 0.01	1.48 ± 0.02	0.29 ± 0.01	58.33 ± 1.25
NND4	66.35 ± 5.13	65.28 ± 65.28	99.98 ± 0.01	1.78 ± 0.06	0.22 ± 0.02	59.67 ± 3.10

Note: GN: Guangdong natural fermentation; HN: Hebei natural fermentation; NN: Hunan natural fermentation; D: day, the number after D represents the fermentation days.

**Table 2:** Statistical analysis of Illumina sequencing data of bacteria from inoculated fermented cowpea samples.

Samples	Ace	Chaos	Coverage (%)	Shannon	Simpson	Sobs
GID0	106.29 ± 24.10	112.2 ± 23.07	99.94 ± 0.01	0.70 ± 0.03	0.66 ± 0.05	85.67 ± 14.38
GID1	81.30 ± 11.83	80.99 ± 10.64	99.96 ± 0.01	0.96 ± 0.07	0.53 ± 0.05	67.00 ± 7.35
GID2	67.78 ± 12.90	54.59 ± 2.31	99.96 ± 0.00	0.56 ± 0.13	0.70 ± 0.09	41.67 ± 6.60
GID3	64.85 ± 8.42	58.57 ± 9.20	99.96 ± 0.01	0.80 ± 0.06	0.52 ± 0.03	47.33 ± 6.55
GID4	86.17 ± 14.37	70.54 ± 3.65	99.96 ± 0.01	0.96 ± 0.08	0.46 ± 0.03	54.00 ± 0.00
HID0	45.02 ± 14.65	44.00 ± 16.35	99.97 ± 0.01	0.50 ± 0.03	0.73 ± 0.03	30.67 ± 7.41
HID1	30.92 ± 2.98	30.31 ± 2.07	99.99 ± 0.00	0.52 ± 0.07	0.72 ± 0.06	27.33 ± 2.62
HID2	29.86 ± 11.52	27.17 ± 5.17	99.98 ± 0.01	0.40 ± 0.05	0.78 ± 0.03	18.67 ± 2.49
HID3	28.72 ± 5.70	26.06 ± 5.44	99.98 ± 0.01	0.49 ± 0.10	0.71 ± 0.07	23.00 ± 4.97
HID4	32.95 ± 9.51	26.67 ± 3.88	99.98 ± 0.01	0.72 ± 0.01	0.50 ± 0.01	22.67 ± 1.89
NID0	58.25 ± 3.55	51.11 ± 3.74	99.96 ± 0.01	0.50 ± 0.10	0.74 ± 0.07	36.67 ± 5.73
NID1	41.06 ± 16.66	31.29 ± 8.34	99.98 ± 0.01	0.45 ± 0.10	0.74 ± 0.08	25.33 ± 5.25
NID2	16.65 ± 1.20	17.00 ± 2.86	99.99 ± 0.01	0.17 ± 0.05	0.93 ± 0.03	12.67 ± 1.25
NID3	40.90 ± 11.33	40.33 ± 18.93	99.98 ± 0.01	0.40 ± 0.16	0.77 ± 0.12	20.00 ± 1.63
NID4	45.91 ± 5.42	32.59 ± 6.31	99.98 ± 0.01	0.57 ± 0.04	0.65 ± 0.04	26.67 ± 5.19

Note: GI: Guangdong inoculated fermentation; HI: Hebei inoculated fermentation; NI: Hunan inoculated fermentation; D: day, the number after D represents the fermentation days.

## Data Availability

The original contributions presented in this study are included in the article. Further inquiries can be directed to the corresponding author.
